# Supplementary figures and images for: Analysis of predicted factors for bronchoalveolar lavage recovery failure: An observational study
Source: PLoS One. 2022 Sep 30;17(9):e0275377. doi: 10.1371/journal.pone.0275377 (PMC9524652; doi:10.1371/journal.pone.0275377)

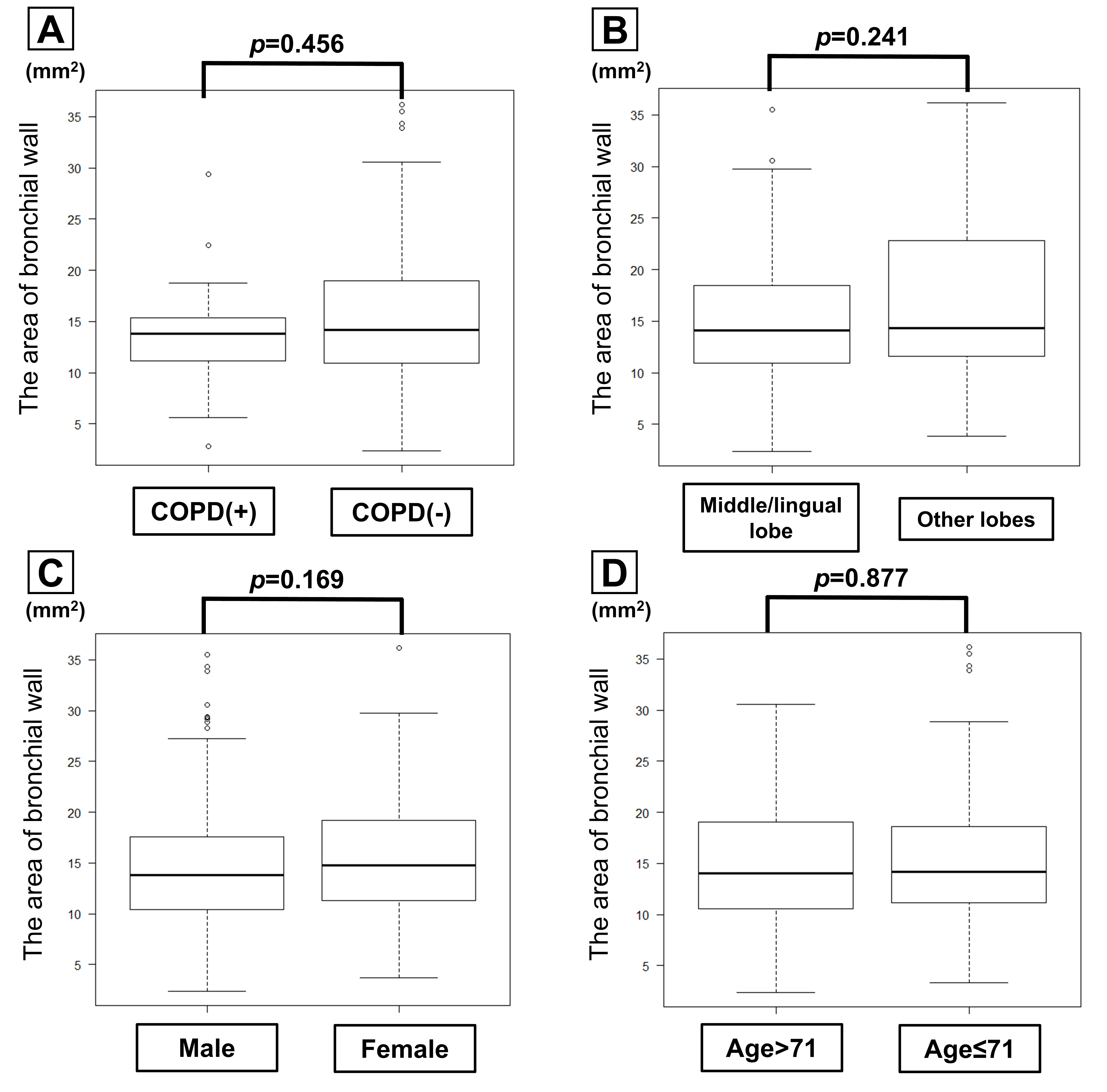

Supplement: S1 Fig — The area of the bronchial wall did not show significant relationships with COPD (having COPD 13.8 mm2 [11.2–15.3] vs. no COPD 14.2 mm2 [10.9–15.3], p = 0.456) in S1A Fig, a target site of BAL (middle/lingual lobe 14.1 mm2 [10.9–18.5] vs. other lobes 14.3 mm2 [11.6–22.7], p = 0.241) in S1B Fig, sex (male 13.8 mm2 [10.4–17.6] vs. female 14.8 mm2 [11.3–19.2], p = 0.169) in S1C Fig, and age (>71 years old median 14.1 mm2 [10.6–19.1] vs. ≤71 years old median 14.2 mm2 [11.2–18.6], p = 0.877) in S1D Fig. (TIF) [file pone.0275377.s001.tif]
