# Supplementary material for: Analysis of predicted factors for bronchoalveolar lavage recovery failure: An observational study
Source: PLoS One. 2022 Sep 30;17(9):e0275377. doi: 10.1371/journal.pone.0275377 (PMC9524652; doi:10.1371/journal.pone.0275377)
Supplement: S1 Table — (DOCX) [file pone.0275377.s002.docx]

Supplemental Table 1. Correlation with the recovery rate of bronchoalveolar lavage fluid

| Variable | n | Correlation coefficient | *p value* |
| --- | --- | --- | --- |
| Age | 338 | -0.131 | 0.016 |
| The amount of cigarettes smoked (pack-year) ^a^ | 326 | -0.212 | <0.001 |
| White blood cell count | 338 | -0.132 | 0.015 |
| C-reactive protein | 338 | -0.042 | 0.444 |
| Lactate dehydrogenase | 336 | 0.100 | 0.068 |
| The area of the bronchial lumen | 338 | 0.023 | 0.672 |
| The area of the bronchial wall | 338 | 0.141 | 0.009 |
| Lung volume affiliated with bronchus, which was a target site for BAL ^a^ | 326 | 0.003 | 0.952 |
| BAL bronchoalveolar lavage fluid  ^a^: n=326 | | | |
