# Supplementary material for: Analysis of predicted factors for bronchoalveolar lavage recovery failure: An observational study
Source: PLoS One. 2022 Sep 30;17(9):e0275377. doi: 10.1371/journal.pone.0275377 (PMC9524652; doi:10.1371/journal.pone.0275377)
Supplement: S2 Table — (DOCX) [file pone.0275377.s003.docx]

Supplemental Table 2. The odds ratio for the BAL recovery rate failure was analysed using Pearson’s chi-squared test.

|  | Odds ratio | 95% Confidence interval | | *p value* |
| --- | --- | --- | --- | --- |
|  |  | Upper limit | Lower limit |  |
| Age >71 years old | 2.60 | 1.20 | 5.94 | 0.013 |
| Male | 2.54 | 1.08 | 6.70 | 0.034 |
| A target site of BAL other than the middle/lingual lobe | 3.34 | 1.36 | 7.80 | 0.004 |
| COPD | 4.93 | 1.55 | 14.4 | 0.002 |
| The area of the bronchial wall <10.6 mm^2^ | 4.39 | 2.02 | 9.59 | <0.001 |
| BAL bronchoalveolar lavage fluid, COPD chronic obstructive pulmonary disease | | | | |
