# Supplementary material for: Analysis of predicted factors for bronchoalveolar lavage recovery failure: An observational study
Source: PLoS One. 2022 Sep 30;17(9):e0275377. doi: 10.1371/journal.pone.0275377 (PMC9524652; doi:10.1371/journal.pone.0275377)
Supplement: Attachment — Submitted filename: Response to Reviewers.docx [file pone.0275377.s005.docx]

**Responses to the editor and the reviewers**

Manuscript ID PONE-D-22-18216
“Analysis of Predicted factors for Bronchoalveolar Lavage Recovery Failure: A Case–Control Study”

**All modifications have been highlighted in the revised manuscript.**

To the editor and the reviewer:

Thank you very much for your constructive comments on our manuscript PONE-D-22-18216. We are pleased to hear that the reviewer found our study intriguing. In response to the journal requests and editer’s comments, we generated text and tables. We carefully studied all comments and made the necessary edits/modifications. The point-by-point responses are listed below. We hope that we have sufficiently addressed the issues raised by the reviewer.

*Journal Requirements:*

*Please review your reference list to ensure that it is complete and correct. If you have cited papers that have been retracted, please include the rationale for doing so in the manuscript text, or remove these references and replace them with relevant current references. Any changes to the reference list should be mentioned in the rebuttal letter that accompanies your revised manuscript. If you need to cite a retracted article, indicate the article’s retracted status in the References list and also include a citation and full reference for the retraction notice.*

Response: Thank you for your comment. We ensured that all references are complete and correct. edited it on references. In addition, we modified them to “Vancouver” style.

*Additional Editor Comments:*

** My original comment was only deleting the "data.xlsx" which contains the individual information of all study participants, not all supplementary materials.*

Response: Thank you for your comment. We re-uploaded two supplemental tables and one supplemental figure, and we described it in manuscript on page 12, line 201, page 15, lines 225-226, page 16, line 245, and on 26, lines 413-425.

** I would like to suggest moving Table 2 to 1 as "Baseline characteristics between the failure group and success group", comparing all variables which was included in the Table 1 of your original submission. Then, Table 1 "Factors affecting the recovery rate of BAL" could be move to Table 2.*

Response: Thank you for your comment. We changed table 1 to table 2, and table 2 to table 1. And the sentence that is related with tables changed the order.

** In Table1, age is missing.*

Response: Thank you for your comment. Age and recovery rate are continuous variables; therefore, we evaluated the correlation between age and recovery rate by using Spearman’s correlation analysis. Please confirmed it on page 12, lines 200-203.
