# Supplementary material for: Analysis of predicted factors for bronchoalveolar lavage recovery failure: An observational study
Source: PLoS One. 2022 Sep 30;17(9):e0275377. doi: 10.1371/journal.pone.0275377 (PMC9524652; doi:10.1371/journal.pone.0275377)
Supplement: Attachment — Submitted filename: Response to Reviewers.docx [file pone.0275377.s006.docx]

**Responses to the editor and the reviewers**

Manuscript ID PONE-D-22-18216
“Analysis of Predicted factors for Bronchoalveolar Lavage Recovery Failure: A Case–Control Study”

**All modifications have been highlighted in the revised manuscript.**

To the editor and the reviewer:

Thank you very much for your constructive comments on our manuscript PONE-D-22-18216. We are pleased to hear that the reviewer found our study intriguing. In response to the journal requests and editer’s comments, we generated text and tables. We carefully studied all comments and made the necessary edits/modifications. The point-by-point responses are listed below. We hope that we have sufficiently addressed the issues raised by the reviewer.

*Journal Requirements:*

*Please review your reference list to ensure that it is complete and correct. If you have cited papers that have been retracted, please include the rationale for doing so in the manuscript text, or remove these references and replace them with relevant current references. Any changes to the reference list should be mentioned in the rebuttal letter that accompanies your revised manuscript. If you need to cite a retracted article, indicate the article’s retracted status in the References list and also include a citation and full reference for the retraction notice.*

Response: Thank you for your comment. We ensured that all references are complete and correct.

*Additional Editor Comments:*

** I believe the authors have addressed almost everything the editor and reviewers raised. I have two minor comments regarding the construction of the Results section.*

** Please correct "The comparisons between the failure group and 194 the success group are shown in Table 2". Comparisons were shown in Table 1. And this sentence would be better to be placed at the first sentence of the 2nd paragraph of Results section. In that paragraph 3rd sentence, please remove "In univariable analysis".*

Response: Thank you for your comment. We changed “The comparisons between the failure group and the success group are shown in Table 2.” to “in Table 1”. We moved “There were 36 patients (10.7%) in the failure group and 302 patients (89.3%) in the success group.” to the end of the 1^st^ paragraph, and “The comparisons between the failure group and the success group are shown in Table 1” moved at the first sentence of the 2^nd^ paragraph. Furthermore, "In univariable analysis" was removed.

** Please separate the paragraph of Results section; one for supplementary Table 1 and another for main Table 2.*

Response: Thank you for your comment. We separated the paragraph related with Table 1 and Table 2.
